# Physiology

**Published:** 1865-08

**Authors:** Austin Flint


PHYSIOLOGY—*By Austin Flint, jr.*—We observe by an advertisement in the *New York Medical Journal* that a new book, on *Human Physiology*, by Dr. Austin Flint, jr. is soon to appear. It embraces a full and comprehensive plan, and will comprise three or four volumes, the first of which is announced for January, 1866.

---

☞ Dr. Clark reported to the Indiana State Medical Society a case of traumatic aneurism of the left carotid artery, which terminated spontaneously in recovery. The report is published in the *Cincinnati Lancet and Observer.*

